# Molecular Characterization, Expression and Functional Analysis of Chicken *STING*

**DOI:** 10.3390/ijms19123706

**Published:** 2018-11-22

**Authors:** Jin-Shan Ran, Jie Jin, Xian-Xian Zhang, Ye Wang, Peng Ren, Jing-Jing Li, Ling-Qian Yin, Zhi-Qiang Li, Dan Lan, Yao-Dong Hu, Yi-Ping Liu

**Affiliations:** 1 Farm Animal Genetic Resources Exploration and Innovation Key Laboratory of Sichuan Province, Sichuan Agricultural University, Chengdu 611130, Sichuan, China; jsran0924@163.com (J.-S.R.); zhangxx5581@163.com (X.-X.Z.); yewangfaith@gmail.com (Y.W.); 15508359925@163.com (P.R.); jingjingli11@163.com (J.-J.L.); 18780058613@163.com (L.-Q.Y.); lzq1998722@163.com (Z.-Q.L.); tianxiaojohn007@163.com (Y.-D.H.); 2 Kunming Primate Research Center, Chinese Academy of Science, Kunming 650223, Yunnan, China; lovejinjie@yahoo.cn; 3 College of Science, Sichuan Agricultural University, Ya’an Sichuan 625014, China; lan.dan1987@163.com

**Keywords:** chicken, *STING*, innate immune, cloning, antiviral response

## Abstract

Innate immunity is an essential line of defense against pathogen invasion which is gained at birth, and the mechanism involved is mainly to identify pathogen-associated molecular patterns through pattern recognition receptors. *STING* (stimulator of interferon genes) is a signal junction molecule that hosts the perception of viral nucleic acids and produces type I interferon response, which plays a crucial role in innate immunity. However, relatively few studies have investigated the molecular characterization, tissue distribution, and potential function of *STING* in chickens. In this study, we cloned the full-length cDNA of chicken *STING* that is composed of 1341 bp. Sequence analyses revealed that *STING* contains a 1140-bp open-reading frame that probably encodes a 379-amino acid protein. Multiple sequence alignments showed that the similarity of the chicken *STING* gene to other birds is higher than that of mammals. Real-time polymerase chain reaction (PCR) assays revealed that *STING* is highly expressed in the spleen, thymus and bursa of fabricious in chickens. Furthermore, we observed that *STING* expression was significantly upregulated both in vitro and in vivo following infection with Newcastle disease virus (NDV). *STING* expression was also significantly upregulated in chicken embryo fibroblasts upon stimulation with poly(I:C) or poly(dA:dT). Taken together, these findings suggest that *STING* plays an important role in antiviral signaling pathways in chickens.

## 1. Introduction

Innate immunity is the general term for the biological defense system of the animal that does not rely on acquired immunity, including physiological barrier, chemical barrier and cells involved in natural immunity. It is an essential line of defense against the invasion of pathogenic microorganisms, which can rapidly produce immune responses to foreign bodies [1]. Innate immunity plays an important role in identifying the invasion of pathogenic microorganisms and establishing effective host defense. When pathogens invade cells, the host recognizes pathogen-associated molecular patterns through a series of pathogen-recognition receptors (PRRs), Toll-like receptors (TLRs), retinoic acid-inducible gene-I-like receptors (RLRs), nucleotide oligomerization doman-like receptors (NLRs), and DNA recognition receptors encoded by the germline genes to perceive the invasion of the pathogen [2,3]. Organism inhibiting and eliminating pathogens are activated by transcription factors to induce the production of interferon type I and pro-inflammatory cytokines through a series of related cell signal transductions [4,5,6].

After recognizing DNA, the DNA receptor transmits the signal to *STING*, a molecule in the endoplasmic reticulum, which then dimerizes rapidly and transfers from the endoplasmic reticulum to the nucleosome via the Golgi apparatus [5]. *STING* is located upstream of *TBK1* (TANK-binding kinase 1) and is capable of responding to type B dsDNA and 5′-3p dsRNA. The aggregation of STING-TBK1 complex driven by DNA stimulation is essential for the activation of *TBK1*, and activated *TBK1* phosphorylated *IRF3* (interferon regulatory factor 3) induces the synthesis of type I interferon to exert various physiological functions such as antiviral and immune regulation. However, during the recognition of RNA virus, *RIG-I* is activated by the virus and transmits the activation signal to MAVS located downstream in the mitochondria. *STING* interacts with MAVS on the mitochondria or MAM structure, and then *STING* is transported to the vesicle structure around the nucleus. As a reaction platform, *STING* recruits a series of immune proteins such as *TBK1* to activate *IRF3*, and the activated *IRF3* forms a dimer into the nucleus to regulate the production of interferon [7,8,9,10]. Overall, *STING* is a signal junction molecule that hosts the perception of viral nucleic acids and produces type I interferon response, which plays a crucial role in innate immunity. 

Human *STING* consists of 379 amino acids, contains four transmembrane domains, and contains a CTD domain at the C-terminus that binds to CDNs in the cytoplasm and recruits downstream signaling proteins [7]. STING has been shown to play an important role in mammalian innate antiviral immunity. It was found that *STING* is involved in RIG-I and MAVS mediated activation of IRF3 and NF-κB in mammals, but which does not participate in MDA5-mediated antiviral pathways [6]. However, subsequent studies have found that there is a type I interferon signaling pathway in chicken cells that does not exist in other animal cells, namely MDA5-MAVS-STING-IRF7-IFNs, which corrected the previous theories that MDA5 cannot induce interferon via *STING*. It is speculated that this signaling pathway plays a very important role in the antiviral innate immune response of chickens [11]. In this study, we cloned the full-length mRNA of chicken *STING* gene, investigated its evolutionary relationships as well as tissue expression patterns, and designed a series of experiments to explore the function of *STING* in the antiviral immunity of poultry.

## 2. Materials and Methods

### 2.1. Chicken, Virus and Tissue Sample Collection

Specific pathogen-free (SPF) chickens were purchased from the Beijing experimental animal center and housed at the experiment farm of Sichuan Agricultural University. All protocols used in this study were approved by the animal ethics committee of Sichuan Agricultural University. Three chickens were killed at the age of 2 weeks. Tissue samples, including the heart, liver, lung, spleen, thymus, bursa of Fabricius, small intestine, kidney, brain, pectoral muscles and leg muscles of each chicken were harvested and immediately snap frozen in liquid nitrogen and then stored at −80 °C. Newcastle disease virus (NDV) standard virulent strain F48E9 and moderate virulent strain Mukteswar were both provided by Huang Yong, professor of the college of veterinary medicine in Sichuan Agricultural University.

### 2.2. Newcastle Disease Virus (NDV) Challenge and Sample Collection

We choose two-week-old chickens for infection with NDV. Twenty two-week-old SPF chickens were randomly divided into uninfected and infected groups. Each of 10 chickens in the infected group was infected intraperitoneally with a total dose of 10^6.0^ 50% embryo infectious dose of NDV in 0.1 mL. The uninfected group was mock-infected with phosphate-buffered saline as a control. At 1, 2 and 4 days post-infection (dpi), immune tissues, including the spleen, thymus and bursa of fabricious, of every killed chicken were collected, snap frozen in liquid nitrogen, and then stored at −80 °C until thawed for RNA isolation.

### 2.3. Cell Culture, *STING* Knockdown, Virus Infection and Transfection of Poly (I:C) and Poly (dA:dT)

CEFs were prepared from 9-day-old SPF chicken embryos and cultured in Dulbecco’s minimal essential medium (DMEM) supplemented with 10% fetal bovine serum, 100 U/mL of penicillin, and 100 μg/mL of streptomycin. Infection of cells with different multiplicities of infection (MOI) of NDV was performed by incubating 500 μL of diluted NDV at 37 °C for 1 h. The supernatant of the diluted virus was replaced with DMEM with 2% FBS, and then incubated for 12 h before cell collection.

The small interfering RNA (siRNA)-mediated knockdown was conducted in 6-well plates. When the CEFS cells reached approximately 70% confluence. Cells in each well were transfected with *STING* siRNAs (Genepharma, Shanghai, China.). Cell transfection was performed using the reagent protocol Lipofectamine 3000 (Invitrogen, Carlsbad, CA, USA). The Lipofectamine 3000 and siRNA were diluted with optim-MEM culture medium. The diluted siRNA and Lipofectamine 3000 were mixed evenly and placed at room temperature for 10 min. The composite was added to the cell culture plate and was mixed in the culture plate. Knocking down efficiency was estimated by quantitative reverse transcription polymerase chain reaction (RT-PCR) of *STING* mRNA.

The double-stranded RNA and DNA used in our study were poly(I:C) and poly(dA:dT), which were purchased from InvivoGen. Transfection of poly(I:C) and poly(dA:dT) was performed using Lipofectamine 2000 reagent according to the manufacturer’s instructions. The poly(I:C), poly(dA:dT), and transfection reagent were diluted with DMEM, respectively. After 5 min, the diluted poly(I:C) and poly(dA:dT) were combined with the diluted transfection reagent, incubated for 20 min, and then added to cell supernatants and incubated for 4h. The cell supernatant was replaced with DMEM supplemented with 10% FBS and then incubated for 9h before cell collection.

### 2.4. Total RNA Extraction and Reverse Transcription

Total RNA was isolated from collected tissues and CEFs using TRIZOL reagent according to the manufacturer’s instructions. Extracted RNA was dissolved in 40 μL of RNase-free water and stored at −80 °C until thawed for reverse transcription. 

RNA integrity and purity was assessed by visual inspection using NanoVue Plus™ spectrophotometer after being electrophoresed in formaldehyde gel. Only RNAs with an A260/A280 ratio between 1.9 and 2.1 were used for cDNA synthesis. Reverse transcription of total RNA was carried out using the PrimeScript™ RT Reagent Kit according to the manufacturer’s instructions. The reaction was performed in a total volume of 20 μL containing 4 μL of 5 × PrimeScript™ Buffer, 1 μL of PrimeScript™ RT Enzyme Mix I, 1 μL of Oligo dT Primer, 1 μL of random hexamers, 11 μL of RNase-free water, and 1 μg of total RNA. The reaction mixture was incubated at 37 °C for 15 min, and 85 °C for 5 s. The cDNA was stored at −20 °C until used for quantitative real-time PCR (qPCR).

### 2.5. Cloning and Sequencing of the Full-Length cDNA for Chicken’s *STING* Gene

According to the predicted *STING* gene mRNA sequence of *Gallus gallus* (accession no.: XM_015293526.1) retrieved from the GenBank database, a pair of primers (Table 1) was designed to amplify the full coding region of chicken *STING* mRNA from the spleen cDNA. The PCR products were separated by electrophoresis on a 3% agarose gel and purified using the E.Z.N.A.^®^ Gel Extraction Kit (Omega Bio-Tek, Norcross, GA, USA). The purified fragments were cloned into the PMD-19T vector, and five clones were selected for sequencing.

We further performed rapid amplification of cDNA ends (RACE) to determine the 5′- and 3′-UTR of chicken TRIM25 cDNA. The 5′ and 3′RACE cDNA were synthesized from spleen RNA using the SMART™ RACE cDNA amplification Kit according to the manufacturer’s instructions. Two sets of gene-specific primers (Table 1) were designed to amplify the 5′- and 3′-UTR of chicken *STING* mRNA based on the sequenced cDNA fragment, respectively. The PCR products of 5′ and 3′ RACE were separated by electrophoresis on a 3% agarose gel and purified using the E.Z.N.A. ^®^ Gel Extraction Kit. The purified fragments were cloned into the pMD-19T vector, and five to 10 clones were selected for sequencing. 

### 2.6. Quantitative Polymerase Chain Reaction (qPCR) Analysis of Chicken *STING*, *IRF7*, *IFN-α* and *IFN-β* mRNA

Relative expression levels of chicken *STING*, *IRF7*, *IFN-α* and *IFN-β* mRNA were measured by qPCR using a CFX-96 qPCR thermal cycle instrument. A dilution series of the standard was used to calibrate each plate in the qPCR assay. Reactions were performed in a volume of 10 μL, which included 1.0 μL of cDNA preparations, 0.5 μL of each specific primer, 5 μL of Ssofast EvaGreen supermix (Bio-Rad, Hercules, CA, USA), and 3.0 μL of ddH2O. The gene-specific primers used in this study are presented in Table 1. The optimum thermal cycling conditions consisted of an initial denaturing step at 98 °C for 2 min, 39 cycles of 98 °C for 2 s, and at the optimal annealing temperature of each primer pair for 20 s. The specificity of amplification was checked by melting curve analyses and 1.5% agarose gel.

### 2.7. Sequence Comparison, Multiple Sequences Alignment, and Statistical Analysis

The open reading frame (ORF) was determined by using ORF Finder (www.ncbi.nlm.nih.gov/gorf/) (April 28th 2018) and translated into the corresponding amino acid sequence. The Blast program (http://www.ncbi.nlm.nih.gov/blast) (April 28th 2018) was used for sequence similarity analyses. Domain prediction was predicted by using the online predicted tools (http://smart.embl.de/) (May 14th 2018). Secondary structure was predicted by using the online predicted tools (https://embnet.vital-it.ch/software/TMPRED_form.html) (May 19th 2018). The three-dimensional (3D) structure analysis was obtained by using the combination of CPHmodel and Pymol1.5. The phylogenetic tree of the mature peptides was constructed in Mega 6.0 by the neighbor-joining method. 

To investigate the tissue distribution of *STING* mRNA in chicken, we chose glyceraldehyde 3-phosphate dehydrogenase (*GAPDH*) as reference genes to determine the expression levels of *STING* mRNA in different tissues. The expression level of *STING*, *IRF7*, *IFN-α* and *IFN-β* mRNA in CEF was calculated relative to the expression of *GAPDH*. All expression data were expressed as means ± standard error (M ± SE). The expression levels of *STING* in vivo of chicken infected with NDV were subjected to t-test of SAS 8.0 software to identify differences between control and NDV-infected groups. The expression level of *STING*, *IRF7*, *IFN-α* and *IFN-β* in CEF response to NDV, poly(I:C) or poly(dA:dT) were subjected into one-way ANOVA of SAS 8.0 software using the Tukey–Kramer method to identify differences between groups. F-values and degrees of freedom (df) in every statistics report were cited in results. Comparisons were considered significant at a probability (*P*) value <0.05.

## 3. Results

### 3.1. Bioinformatics Analysis of *STING*

#### 3.1.1. Cloning and Sequence Analysis of the Chicken *STING*

**Figure 1:**
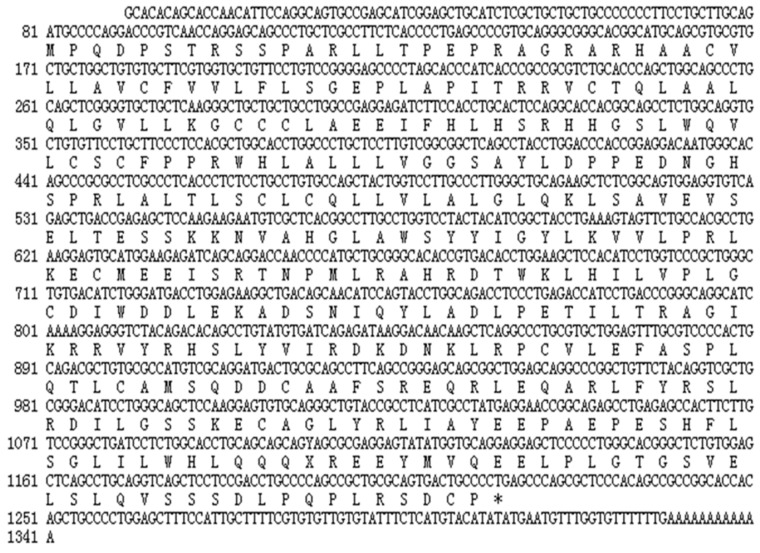
The full-length of chicken *STING* cDNA sequence and the deduced amino acid sequence. The stop codon is shown as “*”. The numbers refer to the nucleotide position.

The full-length chicken *STING* cDNA was found to be 1341bp in length. It consisted of a 1140bp ORF, preceded by 80 bp 5′-UTR and followed by a 121 bp 3′-UTR with a 12 bp poly(A) tail (Figure 1). The chicken *STING* cDNA encoded a predicted 379 amino acid residues protein with an isoelectric point of 6.67 and a molecular weight of 42.6 kDa.

#### 3.1.2. Structure Analysis of Chicken *STING*

**Figure 2:**
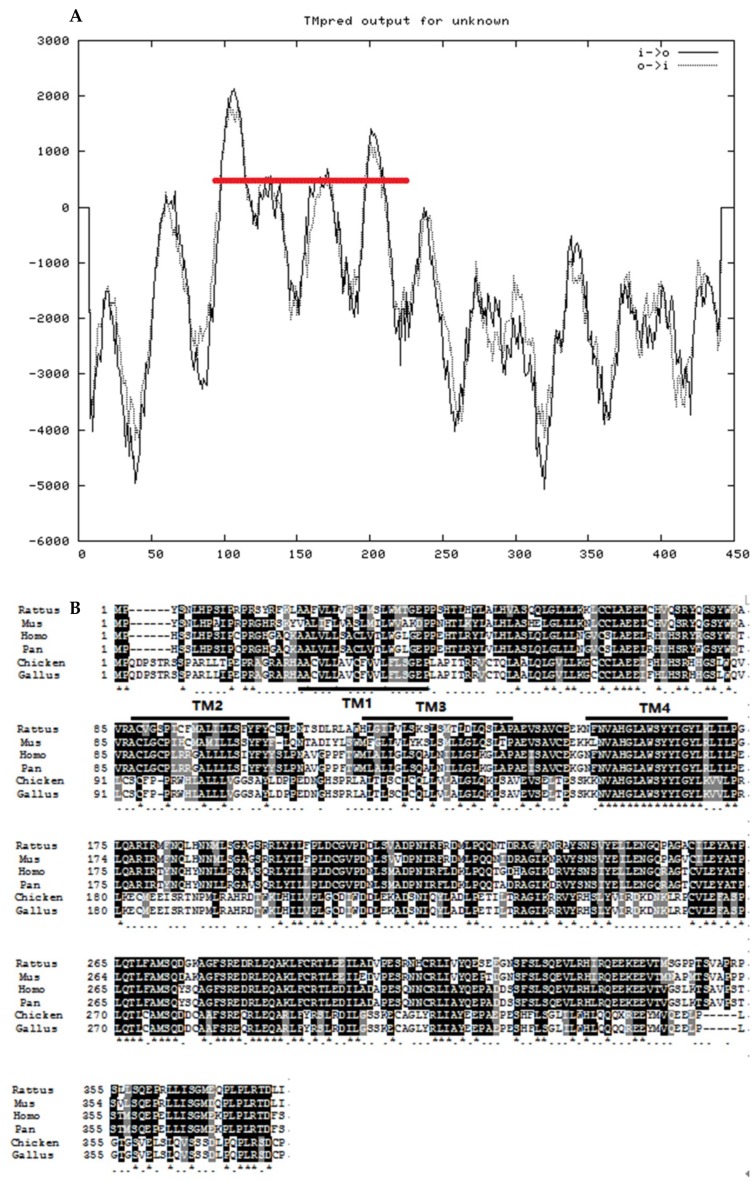
Structures and alignment of predicted amino acid sequence for chicken *STING*. (**A**) Secondary structure and transmembrane tendency prediction of *STING*. The red line means transmembrane domain. (**B**) Multiple alignment of STING amino acid sequences from different species.

According to the prediction of SMART website, we found that the STING protein has a domain TMEM173, which is from amino acid residue 49 to amino acid residue 337. As shown in Figure 2A, the predicted secondary structure of chicken STING consisted of four transmembrane structures (amino acids 27–44, 93–114,126–146, and 158–177). Multiple alignments of STING amino acid sequences from different species are shown in Figure 2B. The black marker is the completely conserved amino acid sequence compared with other species, while the gray marker is 80%–90% conserved amino acid sequence. It can be seen that the conservation of *STING* gene is relatively high in each species.

#### 3.1.3. Comparison and Evolutionary Analyses of Chicken *STING*

**Figure 3:**
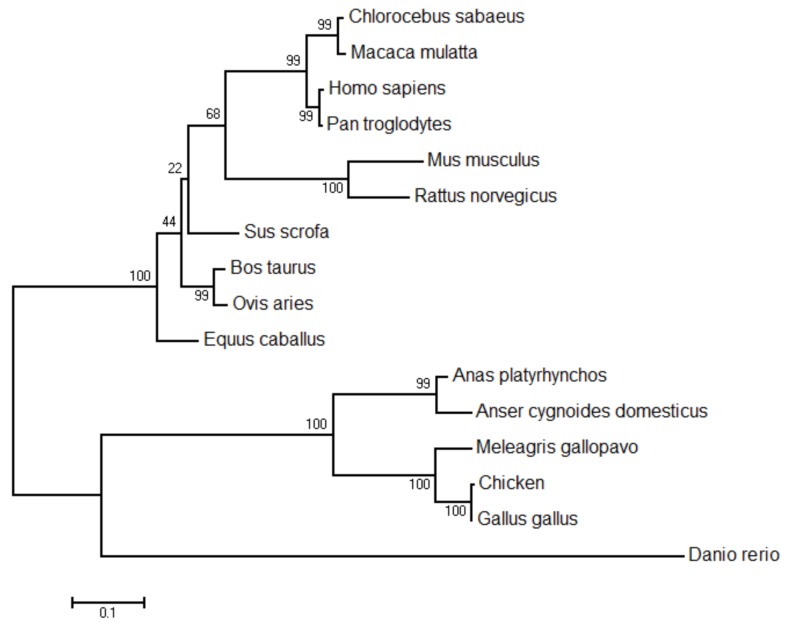
Phylogenetic analysis of chicken *STING*. The scale bar is 0.1.

A comparison of the predicted amino acid sequence of chicken and other species of STING reveals homologies of 99.4%, 69.2%, 67%, 64.8%, 43.4%, 43.7%, 43.5%, 38.7%, and 37.5% for Gallus gallus, Anas platyrhynchos, Anser cygnoides domesticus, Nipponia Nippon, Homo sapiens, Pan troglodytes, Sus scrofa, Mus musculus, and Danio rerio, respectively (Table 2). The results showed that chicken STING has high homology with birds. The phylogenetic tree based on the deduced amino acid sequences revealed that G. gallus STING was grouped into a cluster containing M. gallopavo and other birds STING, and was phylogenetically separated from that of other mammalian species (Figure 3).

### 3.2. Tissue Distribution of *STING* mRNA

**Figure 4:**
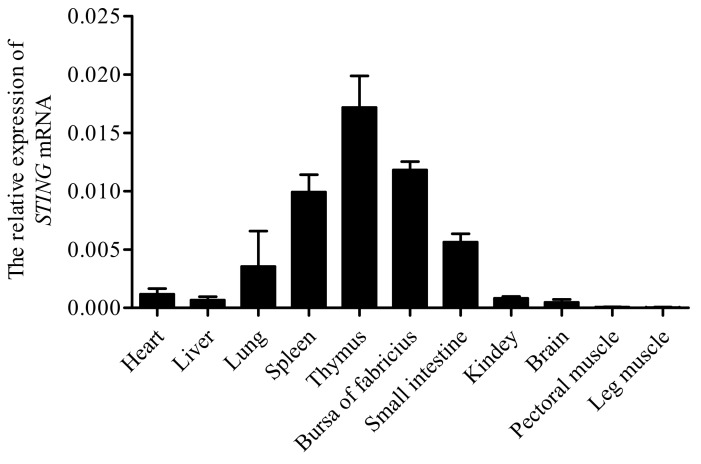
Relative expression levels of *STING* gene in different tissues of chicken were measured. Total RNA from different tissues of three chickens was used to perform the real-time PCR. The expression levels of chicken *STING* gene were normalized to the expression of *GAPDH* gene. The values represent the mean ± SD (*n* = 3).

An expression profile of chicken *STING* was determined by qPCR using *GAPDH* as housekeeping genes. As shown in Figure 4, *STING* was ubiquitously expressed in all examined tissues with the most abundant expression levels in the spleen, bursa of Fabricius, and thymus, followed by the small intestine, lung, kidney, brain, liver, and heart, and relatively quite low expression levels in the pectoral and leg muscle.

### 3.3. Antiviral Function Analysis of *STING*

#### 3.3.1. Interference with *STING* Gene Inhibits the Expression of *IRF7*, *IFN-α* and *IFN-β* Genes in CEFs

**Figure 5:**
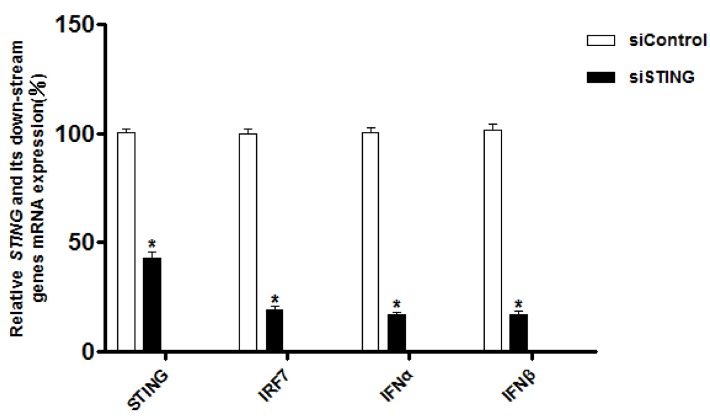
The expression levels of *STING* and its down-stream genes after interference with the *STING* gene. Mean ± standard deviation (SD, *n* = 3) of three biological replicates are shown. (*) *p* < 0.05.

We investigated the potential role of *STING* in MDA5-MAVS-STING-IRF7-IFNs pathway by siRNA-mediated knockdown of *STING* in CEFs. As shown in Figure 5, transfecting these cells with a siRNA targeting *STING* mRNA decreased the expression of endogenous *STING* mRNA by 58.2% as compared to the transfection of a control siRNA. Meanwhile, after transfection of siRNA cells, the expression of *IRF7* gene decreased by 78.6%, and the expression of *IFN-α* and *IFN-β* genes decreased by 80.25% and 80.90%, respectively. These data indicated that *STING* knockdown inhibited the expression of *IRF7*, *IFN-α* and *IFN-β* genes in CEFs.

#### 3.3.2. Expression of *STING* and Its Down-Stream Genes after In Vivo Infection by NDV Strains

**Figure 6:**
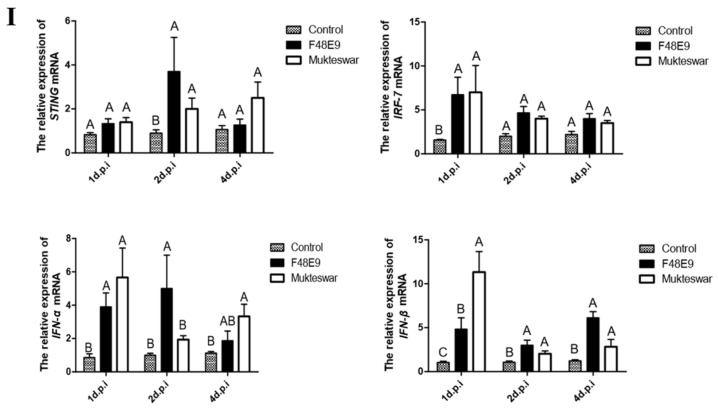

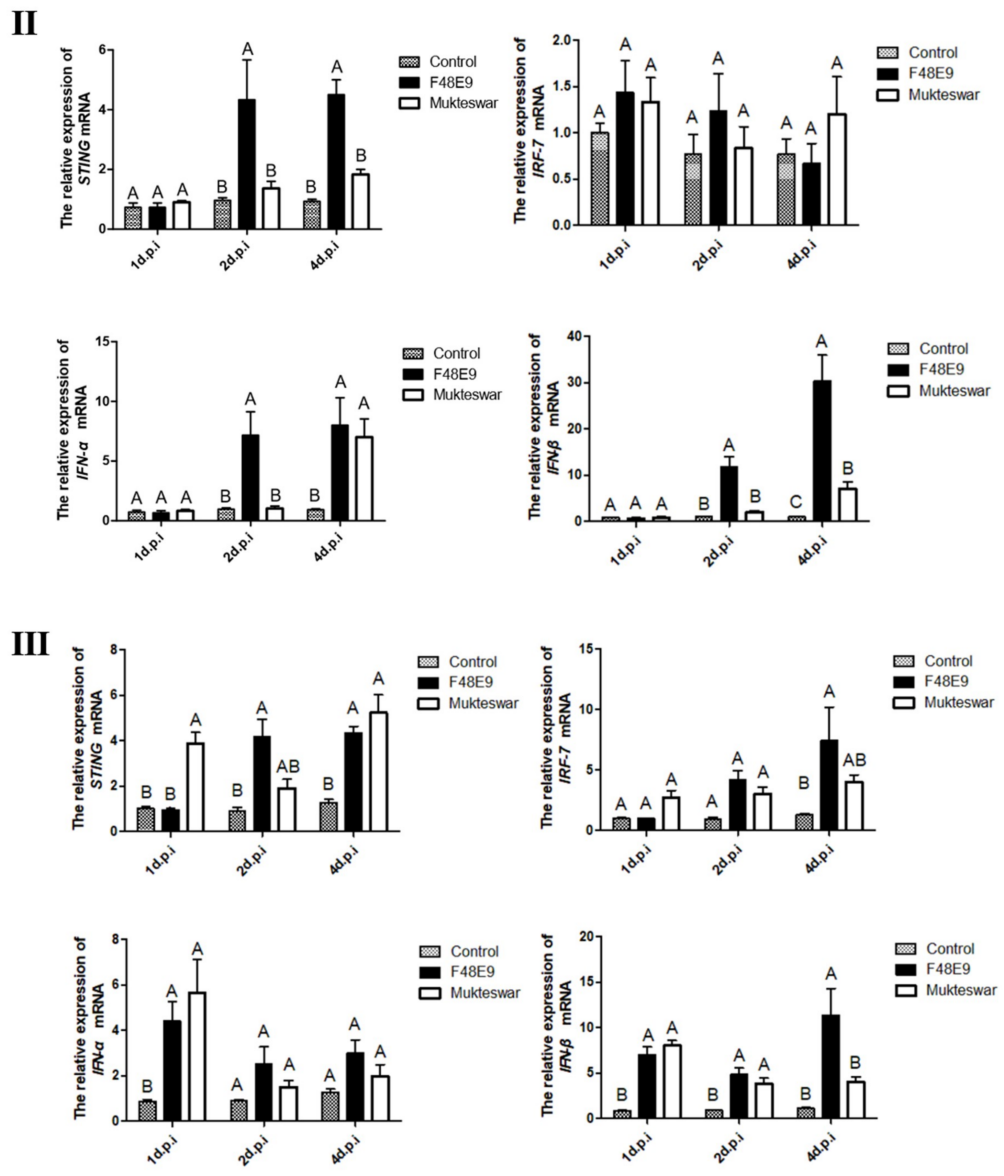
Expression of *STING* and its down-stream genes in the immune-related tissues of Newcastle disease virus (NDV)-infected chickens. (Ⅰ) Specific pathogen-free (SPF) chicken were inoculated with NDV, the expression of *STING* and its down-stream genes in spleen were measured by real-time PCR. (Ⅱ) SPF chicken were inoculated with NDV, the expression of *STING* and its down-stream genes in thymuses of were measured by real-time PCR. (Ⅲ) SPF chicken were inoculated with NDV, the expression of *STING* and its down-stream genes in the bursa of fabricius were measured by real-time PCR. All data shown are the mean ± SD (*n* = 3) and subjected into one-way analysis of variance (ANOVA) to identify differences between groups. Columns on the same dpi sharing completely different capital letters show significant difference (*p* < 0.05).

To establish whether *STING* was involved in antiviral response in vivo, we measured *STING* and its down-stream genes expression levels in the immune-related tissues of chickens after infection with different strains of NDV (Figure 6). The results showed that *STING* and its down-stream genes’ expression levels were upregulated in the spleen, thymus, and bursa of Fabricius of NDV infected chickens at 2 and 4 dpi compared to those in uninfected-control chickens. In addition, the expression of *STING* gene in bursa of Fabricius (Figure 6Ⅲ) on the first day after infection with Mukteswar strain was significantly higher than that of the F48E9 strain, the results indicated that Mukteswar strain triggered innate immune response faster than the F48E9 strain in immune tissue.

#### 3.3.3. Expression of *STING* and its Down-Stream Genes Following CEFs’ Infection with NDV

**Figure 7:**
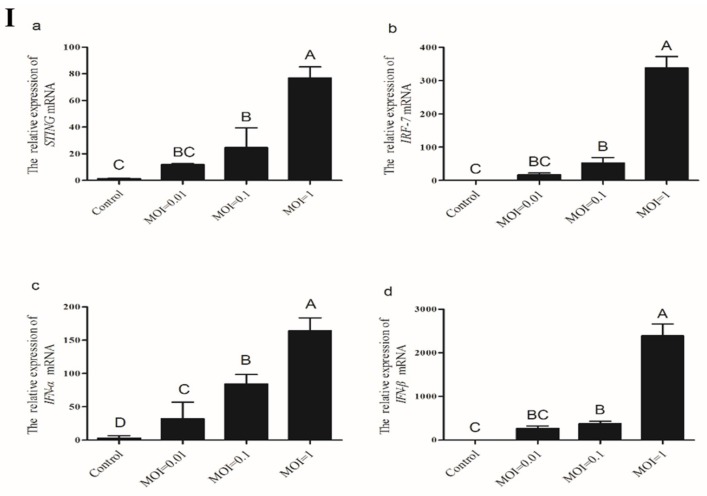

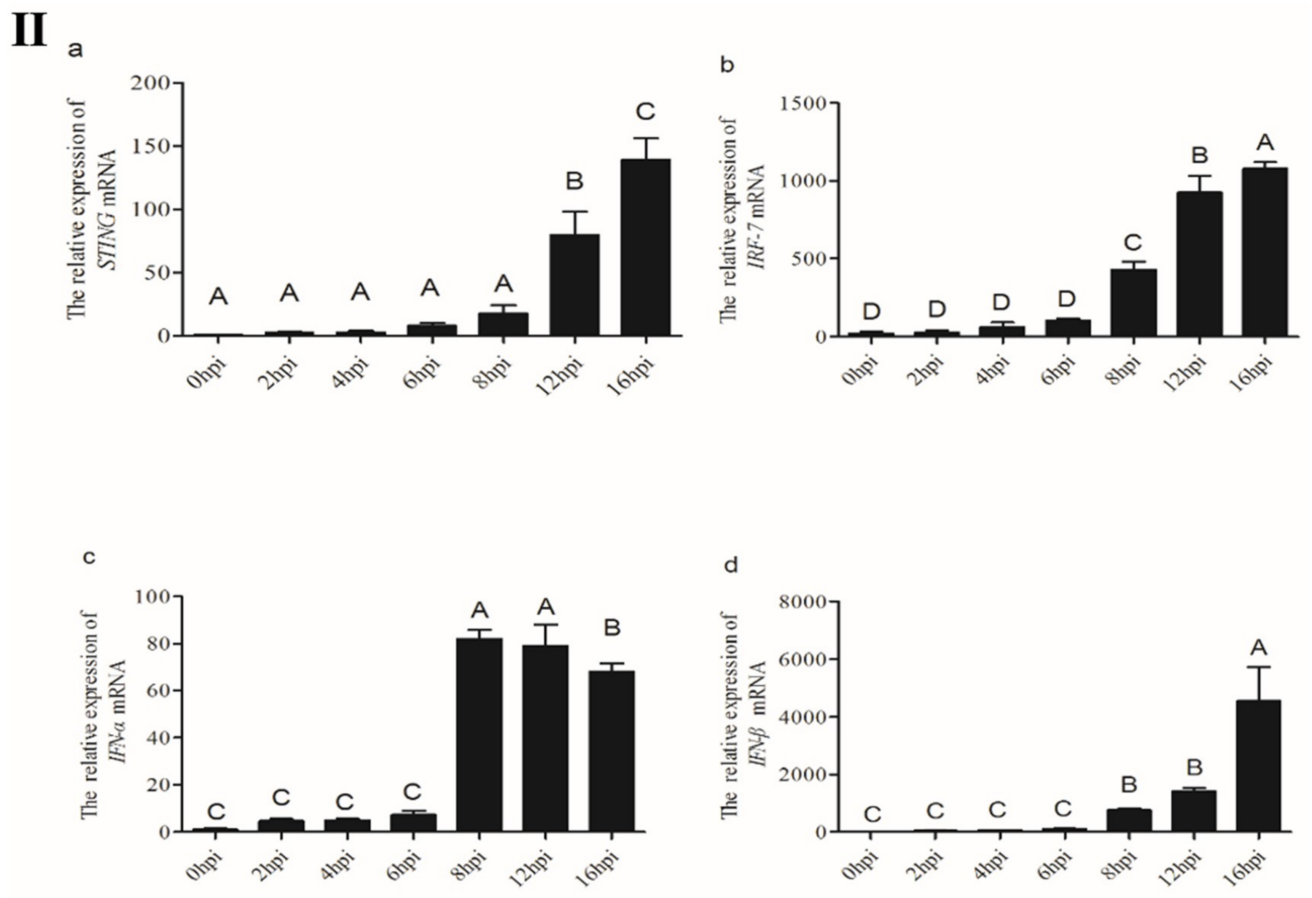
The expression levels of *STING* and its down-stream genes in CEFs after infection with NDV. (Ⅰ) CEFs were infected with different MOIs of NDV and then analyzed for the expression levels of *STING* and its down-stream genes by real-time PCR. (Ⅱ) After infection with NDV, the expression levels of *STING* and its down-stream genes in CEFs were analyzed at different points. All data shown are the mean ± SD (*n* = 3) and subjected into one-way ANOVA to identify differences between groups. Columns sharing completely different capital letters show significant difference (*p* < 0.05).

To characterize whether *STING* was associated with the antiviral immune response of the chicken, *STING* and its down-stream genes expression levels after infection with different doses of Mukteswar strain were analyzed at 16 h post infection (hpi). As shown in Figure 7Ⅰ, *STING* and its downstream genes expression levels in CEFs significantly increased with increasing MOI of NDV from 0.01 to 1. *STING* and its downstream genes expression levels in CEFs infected with an NDV MOI of 1 were significantly higher than that in the control CEFs and those infected with MOIs of 0.01 and 0.1 (*p* < 0.05), respectively.

We then investigated *STING* and its down-stream genes expression levels at different time points in CEFs infected with a MOI of 1 of NDV. The results showed that *STING* and its down-stream genes expression levels in the CEFs increased sharply from 6 hpi and peaked at 16 hpi (Figure 7Ⅱ). Meanwhile, *STING, IRF7*, and *IFN-β* expression levels in NDV-infected CEFs at 16 hpi were significantly higher compared with that at other hpi (*p* < 0.05). Taken together, these results suggest that *STING* might function as an important gene involved in chicken antiviral response.

#### 3.3.4. Induction of Chicken *STING*, *IRF7*, *IFN-α* and *IFN-β* in CEFs Transfected with Poly(I:C) 

**Figure 8:**
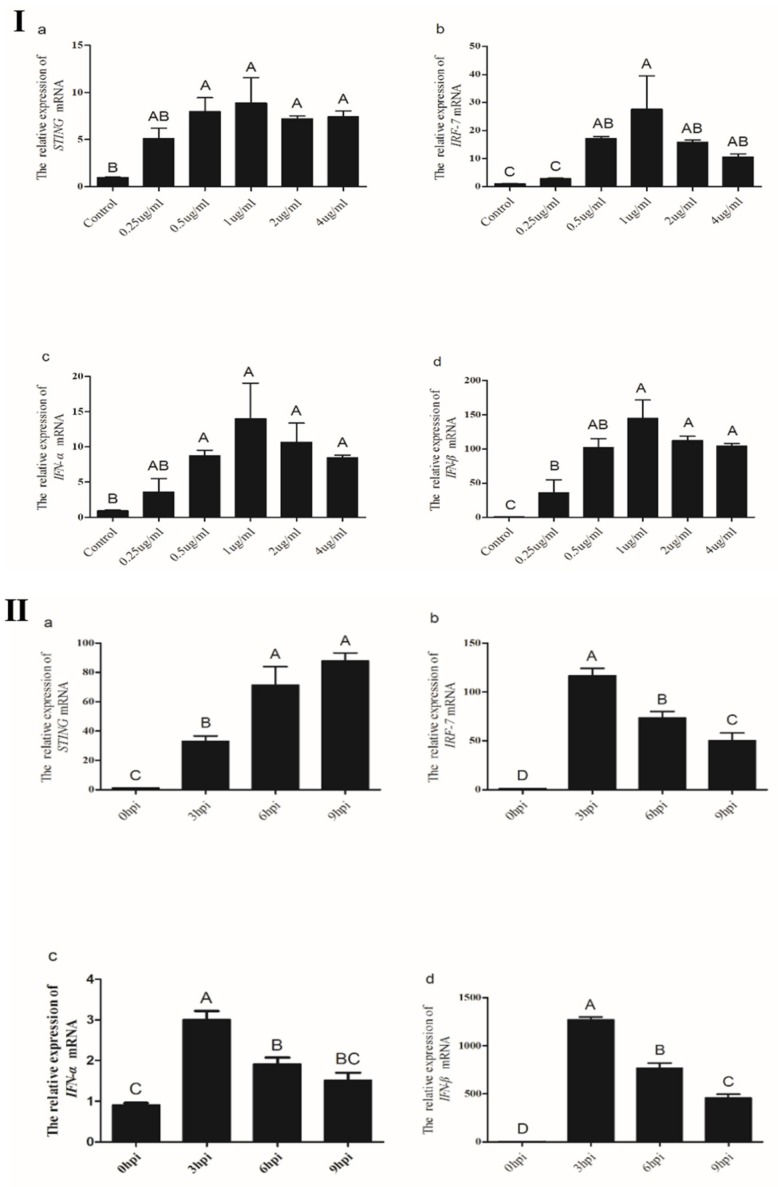
The expression levels of *STING*, *IRF7*, *IFN-α* and *IFN-β* genes in CEFs after transfection with poly(I:C). (Ⅰ) CEF were transfected with different doses of poly(I:C) for 9 h and then analyzed for the expression levels of *STING*, *IRF7*, *IFN-α* and *IFN-β* genes by real-time PCR. (Ⅱ)After transfection with the poly(I:C), the expression levels of *STING*, *IRF7*, *IFN-α* and *IFN-β* genes in CEFs were analyzed at 3, 6, 9 h, respectively. All data shown are the mean ± SD (*n* = 3) and subjected into one-way ANOVA to identify differences between groups. Columns sharing different capital letters show significant difference (*p* < 0.05).

Viral RNA has been shown to immediately trigger antiviral responses in human and murine cells [12]. We determined whether *STING* was involved in the response of chicken cells to dsRNA stimulation. As shown in Figure 8Ⅰ, *STING*, *IRF7*, *IFN-α* and *IFN-β* expression levels in CEFs significantly increased with increasing concentrations of dsRNA mimetic poly(I:C) from 0.25 to 1.0 μg/mL. By contrast, the concentration of poly(I:C) exceeding 1.0 μg/mL resulted in a decrease in *STING*, *IRF7*, *IFN-α* and *IFN-β* expression. 

We also assessed the expression levels of *STING*, *IRF7*, *IFN-α* and *IFN-β* in CEFs after transfection with poly(I:C) at different time points (Figure 8Ⅱ). After transfection with 1.0μg/mL of poly(I:C), the expression levels of *IRF7*, *IFN-α* and *IFN-β* in CEFs peaked at 3h and then gradually decreased; however, the expression trend of the *STING* gene is reversed. Furthermore, expression levels of *STING*, *IRF7*, *IFN-α* and *IFN-β* in CEFs at 3, 6, and 9h post-transfection were significantly higher than in controls (*p* < 0.05), respectively.

#### 3.3.5. Induction of Chicken *STING*, *IRF7*, *IFN-α* and *IFN-β* in CEFs Transfected with Poly(dA:dT)

**Figure 9:**
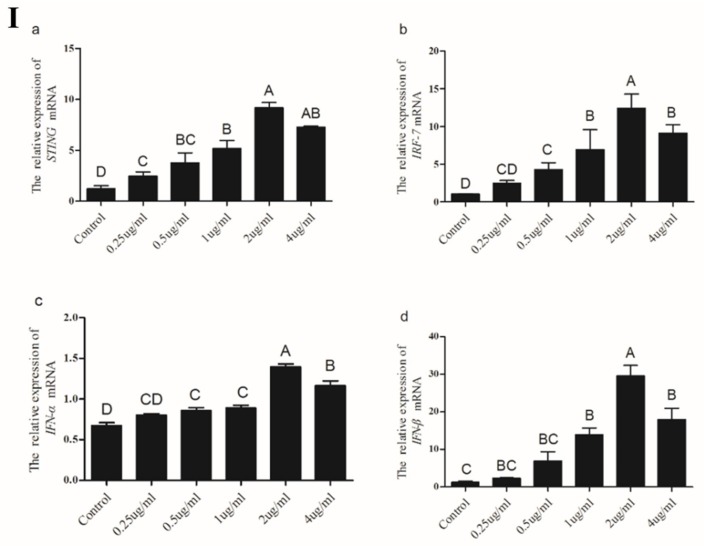

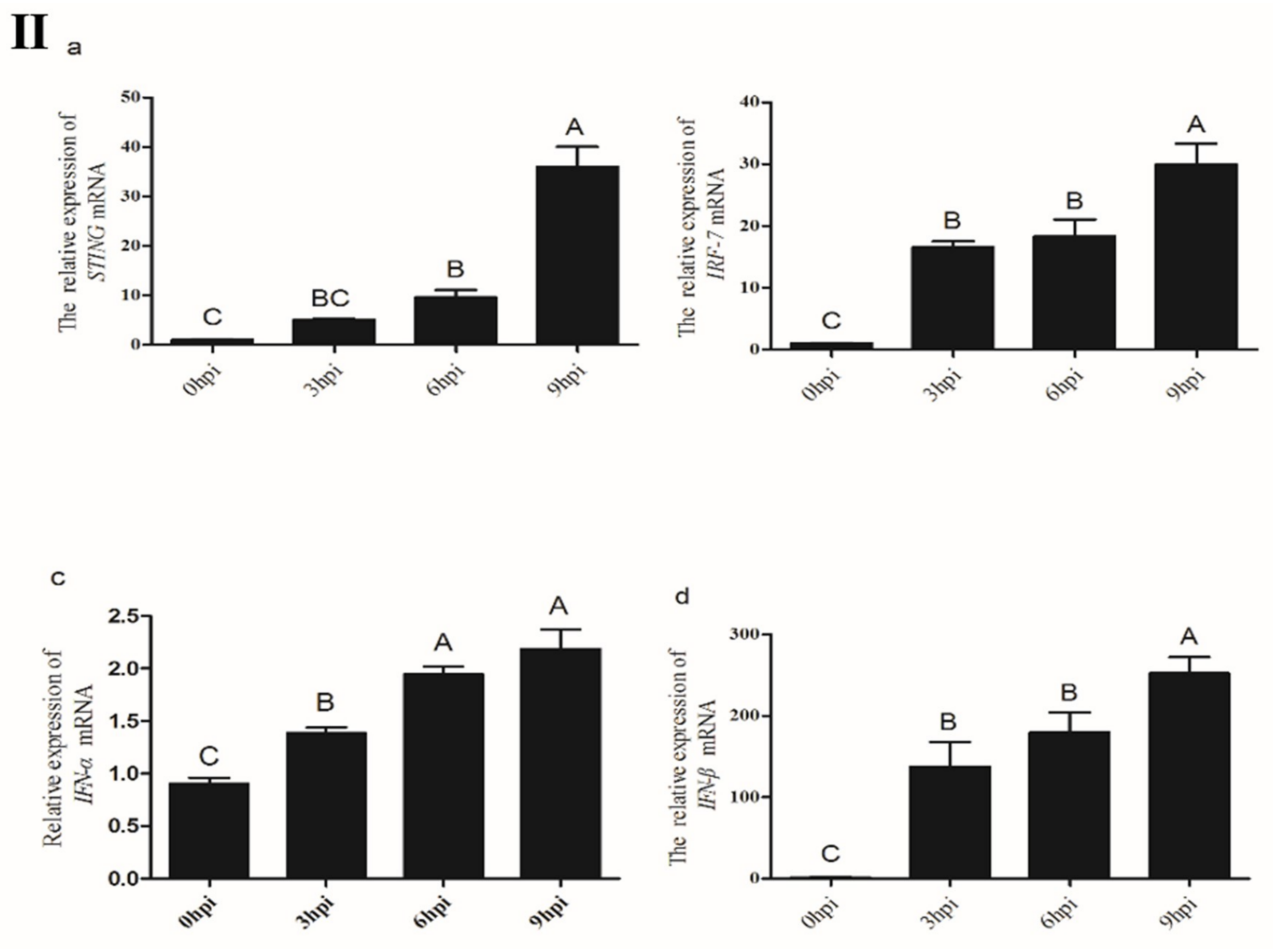
The expression levels of *STING*, *IRF7*, *IFN-α* and *IFN-β* genes in CEFs after transfection with poly(dA:dT). (Ⅰ) CEFs were transfected with different doses of poly(dA:dT) for 9 h and then analyzed for the expression levels of *STING*, *IRF7*, *IFN-α* and *IFN-β* genes by real-time PCR. (Ⅱ)After transfection with the poly(dA:dT), the expression levels of *STING*, *IRF7*, *IFN-α* and *IFN-β* genes in CEFs were analyzed at 3, 6, 9 h, respectively. All data shown are the mean ± SD (*n* = 3) and subjected into one-way ANOVA to identify differences between groups. Columns sharing different capital letters show significant difference (*p* < 0.05).

Viral DNA has also been shown to immediately trigger antiviral responses in human and murine cells [13]. Therefore, we determined whether *STING* was involved in the response of chicken cells to dsDNA stimulation. The results showed that with increasing concentrations of dsDNA-analog poly(dA:dT), *STING*, *IRF7*, *IFN-α* and *IFN-β* expression levels showed an increasing trend in CEFs and peaked at transfection with 2 μg/mL (Figure 9Ⅰ). 

After transfection with 2.0 μg/mL of poly(dA:dT), expression levels of *STING*, *IRF7,* and *IFN-β* in CEFs gradually increased from 3 to 9h post-transfection and peaked at 9 h to levels significantly higher compared to the other two time points (*p* < 0.05) (Figure 9Ⅱ). Meanwhile, expression levels of *IRF7*, *IFN-α*, and *IFN-β* in CEFs at 3, 6, and 9h post-transfection were also significantly higher than those in the control (*p* < 0.05). Overall, these results suggest that *STING* might participate in viral dsRNA, and dsDNA-triggered the antiviral immune response in chicken cells.

## 4. Discussion

Previous studies have shown that the *chSTING* gene is located on chromosome 13 of the chicken, and the chSTING protein mainly exists in the endoplasmic reticulum membrane, but a small amount exists in the mitochondrial membrane [14]. The four transmembrane (TM) functional domains of *chSTING* play an important role in its localization, and the absence of any TM functional domain significantly affects its localization, thereby affecting the activation of IFNβ [8,15]. In this study, we cloned and sequenced full-length chicken *STING* cDNA, which contains a 1140bp ORF, a 80 bp 5′-UTR and a 121 bp 3′-UTR. According to the prediction of SMART website, *chSTING* contains four TM functional domains (transmembrane domains) at the N-terminus. In addition, the *chSTING* gene has six additional amino acids at the N-terminus and lacked five amino acids at the C-terminus. However, whether insertions and deletions of amino acids affect the functionality of *chSTING* requires further study. Sequence alignment showed that *chSTING* had the highest similarity to birds, with similarity to the red junglefowl of 99.4%, humans 43.4%, mice 38.7%, and zebrafish 37.5%. Phylogenetic analysis showed that birds, chickens, and zebrafish belonged to the same subgroup. Mammals include humans, mice, pigs, rats, and horses belonging to another subgroup, and fish belong to the third subgroup.

Exploring the distribution of the *STING* gene can further explain the relationship between immune induction and host pathogens and define biologically infectious diseases in chickens. The *STING* gene with the most abundant expression levels in bacterially invaded immune tissues, such as spleen, thymus and the bursa of fabricius, revealing that the invasion of pathogens can induce *STING* mediated innate immune responses in chickens. We also found that *STING* has a high expression in mucosa- related tissues, such as the small intestine and stomach, because they can directly come into contact with air and food, forming a first barrier to prevent the invasion of foreign pathogens, and making *STING* activated as soon as possible when the pathogen is invaded. Expression profiles of *STING* in different species have been reported previously, such as human [16], mouse [17], pig [18], and rat [19]. Although different species have different tissue expression profiles of *STING*, the study found it most abundant in the spleen which is the largest lymphoid organ in the above species, indicating that *STING* is essential in the immune system.

In mammalian cells, *STING* regulates the transcription and translation of *IFN-β* via *IRF3* [8]. However, more and more studies have reported the natural deletion of the *IRF3* gene in chicken and avian cells, replaced by the *IRF7* gene [20,21]. We speculate that *chIRF7* may function as a mammalian *IRF3* to participate in the *STING* pathway. In our study, the mRNA levels of *chIRF7, IFN-α* and *IFN-β* were down-regulated in *chSTING*-interfering CEFs, and these results preliminarily indicate that *chSTING* may activate *IFN-β* by *chIRF7*.

Newcastle disease virus (NDV) belongs to the *Paramyxoviridae* family and is a single-stranded negative-strand RNA virus with unsegmented genome [22]. NDV can induce interferon synthesis and apoptosis [23,24]. Several studies established critical roles for *STING* in innate immune responses to some RNA viruses. *STING* knockdown impaired Sendai virus-induced and vesicular stomatitis virus (VSV)-induced type I IFN production in human cells [8,9]. In vivo, *STING*-deficient mice are defective in type I IFN production and highly susceptible to lethal infection with VSV, but not EMCV [9]. STING appears to be involved in innate immune responses against RNA viruses in a virus-specific manner. The pathogenicity of NDV strains with different virulence is quite different. The Mukteswar strain belongs to moderate virulence strain, which is commonly used as the standard strain for artificial infection; the F48E9 strain belongs to standard strong virulence strain, which is mostly used as vaccine strain [14]. The results showed that the expression of STING and its down-stream genes were different in different strains. The expression of *STING* and its down-stream genes on the first day after infection in the thymus was not significantly different from that in the control group. In human immunology, the thymus plays a major role in cellular immunity, lymph nodes play a major role in humoral immunity, and the spleen serves as a storage warehouse for peripheral immune cells. However, there is no lymph node in avian immune system, but the bursa of fabricius is the center of humoral immunity. When the pathogen invades the body, the humoral immune response is first initiated, which in turn initiates cellular immunity. Therefore, this discovery reveals that the differential expression of these genes in different tissues may be related to the process of humoral and cellular immunity. In our study, the *STING* gene is involved in innate immune responses to NDV, which is consistent with previous studies on *STING* in mice [25]. In summary, the expression of *STING* and its down-stream genes in the infected group was higher than that in the control group, speculating that *STING* may play an important role in the antiviral pathway.

In human and mouse cells, NDV is mainly recognized by RIG-I [26]. Although RIG-I does not exist in chickens, infection with NDV in spleen, macrophages, spleen leukocytes and chicken embryo fibroblasts can also trigger the production of type I interferons, cytokines and a series of interferon regulatory genes [9,27]. Studies have found that chMDA5 could compensate for the lack of RIG-I by interacting with chSTING to construct the MDA5–STING–IFN-b pathway to activate the anti-RNA virus innate immune response in chicken cells [28,29]. Our results showed that the expression of *STING* and its down-stream genes increased with the increase of MOI titer. The expression level of *IFN-β* was the most significant, indicating that NDV might be affected by its titer. In the same titer of NDV infection with CEFs, *STING* and its down-stream genes began to express significantly after 8 h of infection, allowing for speculation that this may be due to it taking a certain time for NDV to activate the innate immune system. It indicated that *STING* mediated innate immune pathway depends on virus titer and infection time.

Poly(I:C) and poly(dA:dT) are synthetic virus-like nucleic acids that function as PAMPs and immediately cause antiviral responses in human and mouse cells [30]. Poly(I:C) is a synthetic double-stranded RNA virus, it has been previously reported that infection with poly(I:C) activates IRF3 and NF-κB to produce type I interferon [12]. Poly(I:C) is recognized by the body through the pathogen model receptors RIG-I and MDA5. After it is recognized, RIG-I and MDA5 transduce signals to another adaptor protein molecule MAVS in the cytoplasm, and MAVS further activates STING [31,32]. Due to the absence of RIG-I, its function is replaced by MDA5 in chickens [33]. In the present study, after poly(I:C) treatment, *STING* and its down-stream genes were significantly up-regulated, and the expression was highest when poly(I:C) = 1μg/mL. At the same concentration, the *STING* gene was up-regulated with the increase of time, while the down-stream genes were down-regulated. We hypothesize that the signaling pathway of type I interferon is MDA5-MAVS-STING-IRF7-IFNβ in chicken cells, and the down-stream genes may be expressed in large amounts when the virus invades early. Over time, there may be some mechanism to prevent inflammation, leading to the suppression of gene expression, but the mechanism does not target the *STING* gene.

Poly(dA:dT) is a synthetic double-stranded DNA virus that can be directly recognized by RIG-I [13]. In mammals, it is possible to induce the production of type I interferon [34]. In mouse embryonic fibroblasts, macrophages, and dendritic cells, *STING* is essential for the induction of type I interferon production by double-stranded DNA. A series of previous studies have confirmed that *STING* is indispensable for host defense against DNA viruses in the natural immune response triggered by DNA viruses [8,35]. In this study, *STING* and its down-stream genes were significantly expressed after poly(dA:dT) treatment, and the expression was the most abundant when poly(dA:dT) = 2μg/mL. At the same concentration, the expression level of *STING* and its down-stream genes were up-regulated with the increase of time, and *STING* was positively correlated with its down-stream gene expression. In the combination of these results, we implied that the *STING* gene plays a crucial role in the antiviral response triggered by DNA and RNA viruses, but we need to conduct more in-depth research on the molecular mechanisms of these genes at the protein level in the future.

## 5. Conclusions

In the present study, the molecular cloning, nucleotide sequencing, structural, and phylogenetic analyses of the chicken *STING* gene were described for the first time. The ORF of chicken *STING* gene consisted of 1140 bp that probably encoded 379 amino acid residues. Structural analysis indicated that four TM functional domains might play an important role for the function of chicken STING. Real-time PCR results showed that the chicken *STING* gene had high expression levels in immune tissues. Furthermore, this is the first study to observe the increase in expression levels of the chicken *STING* gene both in vitro and in vivo following infection with NDV, and in CEFs transfected with poly (I:C) and poly (dA:dT), respectively. It is indicated that chicken *STING* might be an important component for the antiviral pathway in chickens, and further studies would be helpful to confirm this conclusion.

## Figures and Tables

**Table 1:** Primer sequences for cloning and real-time polymerase chain reaction (PCR).

Gene	Sequence (5′-3′)	Annealing Temperature (°C)	Remarks
*STING*	F:GGACAGTCGCTGTGACCGAGGAT	56.5	Coding region
	R:GCTTGTTGGACCAGTCCTGATG		amplification
5′-RACE	TGCAGACAACTGTCGCAGTAGATC	55	5′GSP
3′-RACE	CCGCCTGGGTAGGAACAGTAGGC	55	3′GSP
*STING*	F:GGACAGTCGCTGTGACCGAGGTT	56.5	Real-time PCR
	R:GCTTGTTGGGCCAGTCCTGATG		
*IRF7*	F:TACACTGAGGACTTGCTGGAGGT	58	Real-time PCR
	R:AAGATGGTGGTCTCCTGATCC		
*IFN-α*	F:CAGGATGCCACCTTCTCTCAC	57.6	Real-time PCR
	R: AGGATGGTGTCGTTGAAGGAG		
*IFN-β*	F:CCTCAACCAGATCCAGCATTAC	57.6	Real-time PCR
	R:CCCAGGTACAAGCACTGTAGTT		
*GAPDH*	F: AGGACCAGGTTGTCTCCTGT	57	Real-time PCR
	R: CCATCAAGTCCACAACACGG		

**Table 2:** The similarity of predicted chicken STING protein with other species.

Species	Uniprot Entry	Identity (%)
*Gallus gallus*	A0A1D5P7Q9	99.4
*Anas platyrhynchos*	A0A071UC76	69.2
*Anser cygnoides domesticus*	A0A053SKR3	67.0
*Nipponia nippon*	A0A091UQ71	64.8
*Homo sapiens*	A0A091FDC9	43.4
*Pan troglodytes*	A0A087C4K5	43.7
*Sus scrofa*	A0A077C6K8	43.5
*Mus musculus*	A0A093SAR9	38.7
*Danio rerio*	K4Q6R6	37.5
